# Supplementary material for: Abnormal Micronutrient Intake Is Associated with the Risk of Periodontitis: A Dose–response Association Study Based on NHANES 2009–2014
Source: Nutrients. 2022 Jun 14;14(12):2466. doi: 10.3390/nu14122466 (PMC9230945; doi:10.3390/nu14122466)
Supplement: Supplementary file 1 [file nutrients-14-02466-s001.zip › File S1.pdf]

Abnormal micronutrient intake is associated with the risk of periodontitis: A dose-response association study based on NHANES

Short title: Micronutrient Intake and Periodontitis

Weiqi Li[1], Qianhui Shang[1,2], Dan Yang[1], Jiakuan Peng[1,2], Hang Zhao*[1], Hao Xu*[1], Qianming Chen[3]

S1 Appendix: Details for eligibility criteria and covariates classification

1.1. eligibility criteria

Inclusion Criteria：

1） Participants with complete periodontal examination data；

2） Participants with complete dietary nutritional intake data；

Exclusion Criteria：

1） Participants with edentulous jaws；

2） Participants with only one dental examination site (not identifiable as periodontitis)；

3） Participants who were breastfeeding at the time of the survey；

4） Participants who were pregnant at the time of the survey.

1.2. Covariates classification

Education is divided into not graduated from high school，High school graduation，Not graduated from college, and college graduation[1, 2]. According to the reference[3], income is categorized into three different levels based on the household income to poverty ratio (IPR) of ≤1.30, 1.31-3.50 and >3.50. physical activity, Participants were determined to be physically active on a

regular basis based on whether they had 150 minutes of moderate to high physical activity per week[4]. Never smokers (<100 cigarettes in their lifetime and currently do not smoke), former smokers (≥ 100 cigarettes in their lifetime and currently do not smoke), and active smokers (≥ 100 cigarettes in their lifetime and currently smoke). A participant is considered as a drinker if he or she consumes ≥ 12 alcoholic beverages in a year or has had ≥ 12 drinks in his or her lifetime[5]. We use the measured Body Mass Index (BMI) as a judgment of obesity. Self-reported diabetes or glycated hemoglobin was > 6.5% was defined as a diabetic patient (https://www.ncbi.nlm.nih.gov/pmc/articles/PMC2797388/). Hypertensive patients were based on SBP ≥140 mm Hg and/or DBP ≥90 mm Hg and participants taking antihypertensive medication. According to the criteria provided by the US National Library of Medicine[6], Total Cholesterol Level ≥240mg/dL or Low-density lipoprotein (LDL) Cholesterol Level ≥130 mg/dL are hyperlipidemic patients (https://medlineplus.gov/lab-tests/).

1. Wright, D.M., et al., *Association between diet and periodontitis: a cross-sectional study of 10,000 NHANES participants.* Am J Clin Nutr, 2020. **112**(6): p. 1485-1491.
2. Bidinotto, A.B., et al., *Investigation of direct and indirect association of ultra-processed food intake and periodontitis.* J Periodontol, 2021.
3. Lan, T., et al., *BMI modifies the association between dietary intake and serum levels of PCBs.* Environ Int, 2021. **156**: p. 106626.
4. Chen, F., et al., *Association Among Dietary Supplement Use, Nutrient Intake, and Mortality Among U.S. Adults: A Cohort Study.* Ann Intern Med, 2019. **170**(9): p. 604-613.
5. Arora, M., et al., *Association of environmental cadmium exposure with periodontal disease in U.S. adults.* Environ Health Perspect, 2009. **117**(5): p. 739-44.
6. Brook, R.D. and S. Rajagopalan, *2017 ACC/AHA/AAPA/ABC/ACPM/AGS/APhA/ASH/ASPC/NMA/PCNA Guideline for the Prevention, Detection, Evaluation, and Management of High Blood Pressure in Adults. A report of the American College of Cardiology/American Heart Association Task Force on Clinical Practice Guidelines.* J Am Soc Hypertens, 2018. **12**(3): p. 238.
