# Supplementary material for: Abnormal Micronutrient Intake Is Associated with the Risk of Periodontitis: A Dose–response Association Study Based on NHANES 2009–2014
Source: Nutrients. 2022 Jun 14;14(12):2466. doi: 10.3390/nu14122466 (PMC9230945; doi:10.3390/nu14122466)
Supplement: Supplementary file 1 [file nutrients-14-02466-s001.zip › Table S1.pdf]

Table S1. The classification criteria for inadequate intake

Recommended Dietary Allowance		
Nutrition	Measurement:	Deficiency
Vitamin_A	µg RAEa/day	
Gender	Age	
Males	All	<900
Females	All	<700
Vitamin_B1 (Thiamin)	mg/day	
Gender	Age	
Males	All	<1.2
Females	All	<1.1
Vitamin_B2 (Riboflavin)	mg/day	
Gender	Age	
Males	All	<1.3
Females	All	<1.1
Vitamin_B3 (Niacin)	mg/day	
Gender	Age	
Males	All	<16
Females	All	<14
Vitamin_B6	mg/day	
Gender	Age	
Males	≤50	<1.3
	>50	<1.7
Females	≤50	<1.3
	>50	<1.5
Vitamin_B9 (Total_folate)	µg/day	
Gender	Age	
All	All	<400
Vitamin_B12	µg/day	
Gender	Age	
All	All	<2.4
Vitamin_C	mg/day	
Gender	Age	
Males	All	<90
Females	All	<75

Vitamin_E	mg/day	
Gender	Age	
All	All	<15

Copper	mg/day	
Gender	Age	
All	All	<0.9

Iron	mg/day	
Gender	Age	
Males	≤50	<8
	>50	<8
Females	≤50	<18
	>50	<8

Magnesium	mg/day	
Gender	Age	
Males	All	<420
Females	All	<320

Phosphorus	mg/day	
Gender	Age	
All	All	<700

Selenium	μg/day	
Gender	Age	
All	All	<55

Zinc	mg/day	
Gender	Age	
Males	All	<11
Females	All	<8

Carbohydrate	g/day	
Gender	Age	
All	All	<130

Protein	g/day	
Gender	Age	
Males	All	<56
Females	All	<46

Adequate Intake

Nutrition	Measurement:	Deficiency

Vitamin_D	µg/day	
Gender	Age	
All	≤50	<5
All	50-70	<10
All	>70	<15

Vitamin_K	µg/day	
Gender	Age	
Males	All	<120
Females	All	<90

Calcium	µg/day	
Gender	Age	
All	≤50	<1000
All	>50	<1200

Potassium	mg/day	
Gender	Age	
All	All	<4700

Sodium	mg/day	
Gender	Age	
All	≤50	<1500
All	50-70	<1300
All	>70	<1200

Dietary_fiber	g/day	
Gender	Age	
Males	≤50	<25
	>50	<38
Females	≤50	<21
	>50	<30

Total_choline	mg/day	
Gender	Age	
Males	All	<550
Females	All	<425
