# Supplementary material for: Abnormal Micronutrient Intake Is Associated with the Risk of Periodontitis: A Dose–response Association Study Based on NHANES 2009–2014
Source: Nutrients. 2022 Jun 14;14(12):2466. doi: 10.3390/nu14122466 (PMC9230945; doi:10.3390/nu14122466)
Supplement: Supplementary file 1 [file nutrients-14-02466-s001.zip › Table S2.pdf]

Table S2. Details of adjusted covariates for each model

Model	Logistic	PSM
Model0	glm(PD~ Nutrition)	NA
Model1	glm(PD~ Nutrition+ Age+ Gender+Education level+ income+ physical activity+ smoking status+ alcohol use+ BMI+ diabetes+ hypertension+ hyperlipidemia)	NA
Model2	glm(PD~ Nutrition+ physical activity+ smoking status+ alcohol use)	Psm(PD~ Age+ Gender+Education level+ income+ BMI+ diabetes + hypertension+ hyperlipidemia)
Model3	glm(PD~ Nutrition)	Psm(PD~ Age+Gender+Education level+ income+ physical activity+ smoking status+ alcohol use+ BMI+ diabetes+ hypertension+ hyperlipidemia)
