# Supplementary material for: Abnormal Micronutrient Intake Is Associated with the Risk of Periodontitis: A Dose–response Association Study Based on NHANES 2009–2014
Source: Nutrients. 2022 Jun 14;14(12):2466. doi: 10.3390/nu14122466 (PMC9230945; doi:10.3390/nu14122466)
Supplement: Supplementary file 1 [file nutrients-14-02466-s001.zip › Table S3.pdf]

Table S3. Details for the micronutrient's intake unit's adjustment

Nutrition	unadjusted	adjusted
Vitamins		
Vitamin_A	1μg	1mg
Vitamin_B1	1mg	1mg
Vitamin_B2	1mg	1mg
Vitamin_B3	1mg	100mg
Vitamin_B6	1mg	1mg
Vitamin_B9	1μg	100μg
Vitamin_B12	1μg	10μg
Vitamin_C	1mg	100mg
Vitamin_D	1μg	10μg
Vitamin_E	1mg	1mg
Vitamin_K	1μg	100μg
Minerals		
Calcium	1mg	100mg
Copper	1mg	1mg
Iron	1mg	10mg
Magnesium	1mg	100mg
Phosphorus	1mg	1g
Potassium	1mg	1g
Selenium	1μg	100μg
Sodium	1mg	1g
Zinc	1mg	10mg
