# Supplementary material for: Abnormal Micronutrient Intake Is Associated with the Risk of Periodontitis: A Dose–response Association Study Based on NHANES 2009–2014
Source: Nutrients. 2022 Jun 14;14(12):2466. doi: 10.3390/nu14122466 (PMC9230945; doi:10.3390/nu14122466)
Supplement: Supplementary file 1 [file nutrients-14-02466-s001.zip › Table S4.pdf]

Table S4. Characteristic of continuous variables of model 3

	Overall [a]	Moderate/Severe periodontitis	No/Mild periodontitis	*P*
No.	5530	2765	2765	
Vitamin_A (/1mg)	0.53 [0.34, 0.80]	0.55 [0.35, 0.83]	0.51 [0.32, 0.77]	<0.001
Vitamin_B1 (/1mg)	1.51 [1.13, 2.05]	1.52 [1.15, 2.06]	1.50 [1.10, 2.03]	0.047
Vitamin_B2 (/1mg)	1.92 [1.39, 2.60]	2.00 [1.43, 2.69]	1.85 [1.36, 2.51]	<0.001
Vitamin_B3 (/10mg)	2.32 [1.73, 3.10]	2.33 [1.76, 3.10]	2.32 [1.70, 3.08]	0.506
Vitamin_B6 (/1mg)	1.93 [1.40, 2.63]	1.95 [1.42, 2.64]	1.92 [1.39, 2.62]	0.239
Vitamin_B9 (/100μg)	3.64 [2.64, 5.00]	3.69 [2.67, 5.04]	3.60 [2.61, 4.94]	0.199
Vitamin_B12 (/10μg)	0.45 [0.29, 0.69]	0.46 [0.29, 0.72]	0.44 [0.28, 0.67]	0.016
Vitamin_C (/100mg)	0.70 [0.35, 1.22]	0.69 [0.35, 1.20]	0.71 [0.34, 1.25]	0.379
Vitamin_D (/10μg)	0.41 [0.23, 0.67]	0.41 [0.23, 0.69]	0.40 [0.22, 0.66]	0.123
Vitamin_E (/1mg)	7.01 [4.84, 9.98]	7.27 [5.12, 10.21]	6.77 [4.63, 9.71]	<0.001
Vitamin_K (/100μg)	0.76 [0.46, 1.31]	0.78 [0.47, 1.34]	0.74 [0.44, 1.28]	0.014
Calcium (/1g)	8.35 [5.96, 11.75]	8.61 [6.15, 11.95]	8.14 [5.81, 11.52]	0.001
Copper (/1mg)	1.14 [0.88, 1.53]	1.16 [0.89, 1.54]	1.12 [0.86, 1.52]	0.035
Iron (/10mg)	1.36 [1.01, 1.83]	1.38 [1.02, 1.86]	1.35 [0.99, 1.82]	0.086
Magnesium (/100mg)	2.80 [2.14, 3.66]	2.84 [2.19, 3.70]	2.77 [2.10, 3.62]	0.018
Phosphorus (/1g)	1.26 [0.97, 1.64]	1.28 [0.99, 1.66]	1.24 [0.96, 1.62]	0.018
Potassium (/1g)	2.55 [1.96, 3.27]	2.56 [2.01, 3.29]	2.54 [1.92, 3.25]	0.141
Selenium (/100μg)	1.05 [0.77, 1.38]	1.05 [0.78, 1.37]	1.05 [0.77, 1.40]	0.79
Sodium (/1g)	3.17 [2.38, 4.14]	3.20 [2.39, 4.16]	3.15 [2.37, 4.12]	0.292
Zinc (/10mg)	1.02 [0.75, 1.39]	1.03 [0.76, 1.41]	1.02 [0.75, 1.37]	0.038

a: Median [quartile] of nutrient intake; b: Rank sum test was used for all classified variables.
