# Supplementary material for: Abnormal Micronutrient Intake Is Associated with the Risk of Periodontitis: A Dose–response Association Study Based on NHANES 2009–2014
Source: Nutrients. 2022 Jun 14;14(12):2466. doi: 10.3390/nu14122466 (PMC9230945; doi:10.3390/nu14122466)
Supplement: Supplementary file 1 [file nutrients-14-02466-s001.zip › Table S5.pdf]

Table S5. Characteristic of categorical variables of model 3

	Level [a]	Overall [b]	Moderate/Severe periodontitis	No/Mild periodontitis	*P* [c]
No.		5530	2765	2765	
PD_dia (%)	Moderate/Severe	2765 (50.0)	2765 (100.0)	0 (0.0)	<0.001
	No/Mild	2765 (50.0)	0 (0.0)	2765 (100.0)	
Vitamin_A (%)	Recommend	1359 (24.6)	728 (26.3)	631 (22.8)	0.003
	Insufficient	4171 (75.4)	2037 (73.7)	2134 (77.2)	
Vitamin_B1 (%)	Recommend	4112 (74.4)	2089 (75.6)	2023 (73.2)	0.045
	Insufficient	1418 (25.6)	676 (24.4)	742 (26.8)	
Vitamin_B2 (%)	Recommend	4675 (84.5)	2370 (85.7)	2305 (83.4)	0.017
	Insufficient	855 (15.5)	395 (14.3)	460 (16.6)	
Vitamin_B3 (%)	Recommend	4708 (85.1)	2368 (85.6)	2340 (84.6)	0.307
	Insufficient	822 (14.9)	397 (14.4)	425 (15.4)	
Vitamin_B6 (%)	Recommend	3914 (70.8)	1966 (71.1)	1948 (70.5)	0.615
	Insufficient	1616 (29.2)	799 (28.9)	817 (29.5)	
Vitamin_B9 (%)	Recommend	2333 (42.2)	1176 (42.5)	1157 (41.8)	0.624
	Insufficient	3197 (57.8)	1589 (57.5)	1608 (58.2)	
Vitamin_B12 (%)	Recommend	4548 (82.2)	2297 (83.1)	2251 (81.4)	0.113
	Insufficient	982 (17.8)	468 (16.9)	514 (18.6)	
Vitamin_C (%)	Recommend	2382 (43.1)	1161 (42.0)	1221 (44.2)	0.109
	Insufficient	3148 (56.9)	1604 (58.0)	1544 (55.8)	
Vitamin_D (%)	Adequate	1221 (22.1)	632 (22.9)	589 (21.3)	0.173
	Insufficient	4309 (77.9)	2133 (77.1)	2176 (78.7)	
Vitamin_E (%)	Recommend	435 (7.9)	243 (8.8)	192 (6.9)	0.013
	Insufficient	5095 (92.1)	2522 (91.2)	2573 (93.1)	
Vitamin_K (%)	Adequate	1911 (34.6)	986 (35.7)	925 (33.5)	0.090
	Insufficient	3619 (65.4)	1779 (64.3)	1840 (66.5)	
Calcium (%)	Adequate	1625 (29.4)	844 (30.5)	781 (28.2)	0.067
	Insufficient	3905 (70.6)	1921 (69.5)	1984 (71.8)	
Copper (%)	Recommend	4021 (72.7)	2043 (73.9)	1978 (71.5)	0.053
	Insufficient	1509 (27.3)	722 (26.1)	787 (28.5)	
Iron (%)	Recommend	4178 (75.6)	2117 (76.6)	2061 (74.5)	0.085
	Insufficient	1352 (24.4)	648 (23.4)	704 (25.5)	
Magnesium (%)	Recommend	1292 (23.4)	668 (24.2)	624 (22.6)	0.172
	Insufficient	4238 (76.6)	2097 (75.8)	2141 (77.4)	

Phosphorus (%)	Recommend	5066 (91.6)	2551 (92.3)	2515 (91.0)	0.090
	Insufficient	464 (8.4)	214 (7.7)	250 (9.0)	
Potassium (%)	Adequate	272 (4.9)	132 (4.8)	140 (5.1)	0.663
	Insufficient	5258 (95.1)	2633 (95.2)	2625 (94.9)	
Selenium (%)	Recommend	5072 (91.7)	2539 (91.8)	2533 (91.6)	0.807
	Insufficient	458 (8.3)	226 (8.2)	232 (8.4)	
Sodium (%)	Adequate	5357 (96.9)	2691 (97.3)	2666 (96.4)	0.064
	Insufficient	173 (3.1)	74 (2.7)	99 (3.6)	
Zinc (%)	Recommend	3258 (58.9)	1654 (59.8)	1604 (58.0)	0.180
	Insufficient	2272 (41.1)	1111 (40.2)	1161 (42.0)	

a: Variables that provide Recommended Dietary Allowance(RDA) are divided into "Recommend" and "Insufficient" groups according to whether RDA is reached. Variables that provide Adequate Intake(AI) are divided into "Adequate" and "Insufficient" groups according to whether AI is reached;

b: Number of people with adequate and insufficient nutrient intake (percentage);

c: Chi square test was used for all categorical variables.
