# Supplementary figures and images for: Abnormal Micronutrient Intake Is Associated with the Risk of Periodontitis: A Dose–response Association Study Based on NHANES 2009–2014
Source: Nutrients. 2022 Jun 14;14(12):2466. doi: 10.3390/nu14122466 (PMC9230945; doi:10.3390/nu14122466)

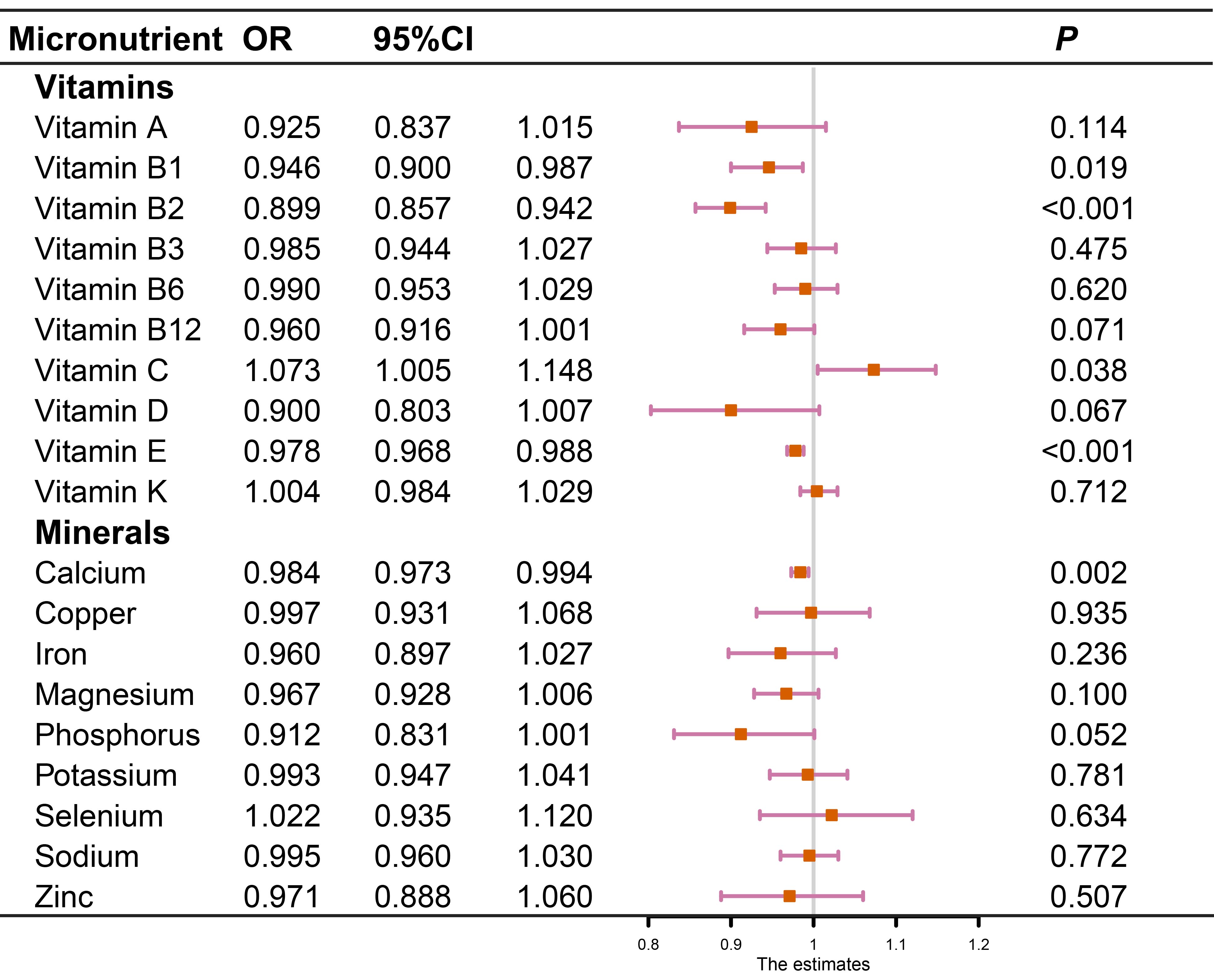

Supplement: Supplementary file 1 [file nutrients-14-02466-s001.zip › Figure S1.jpg]

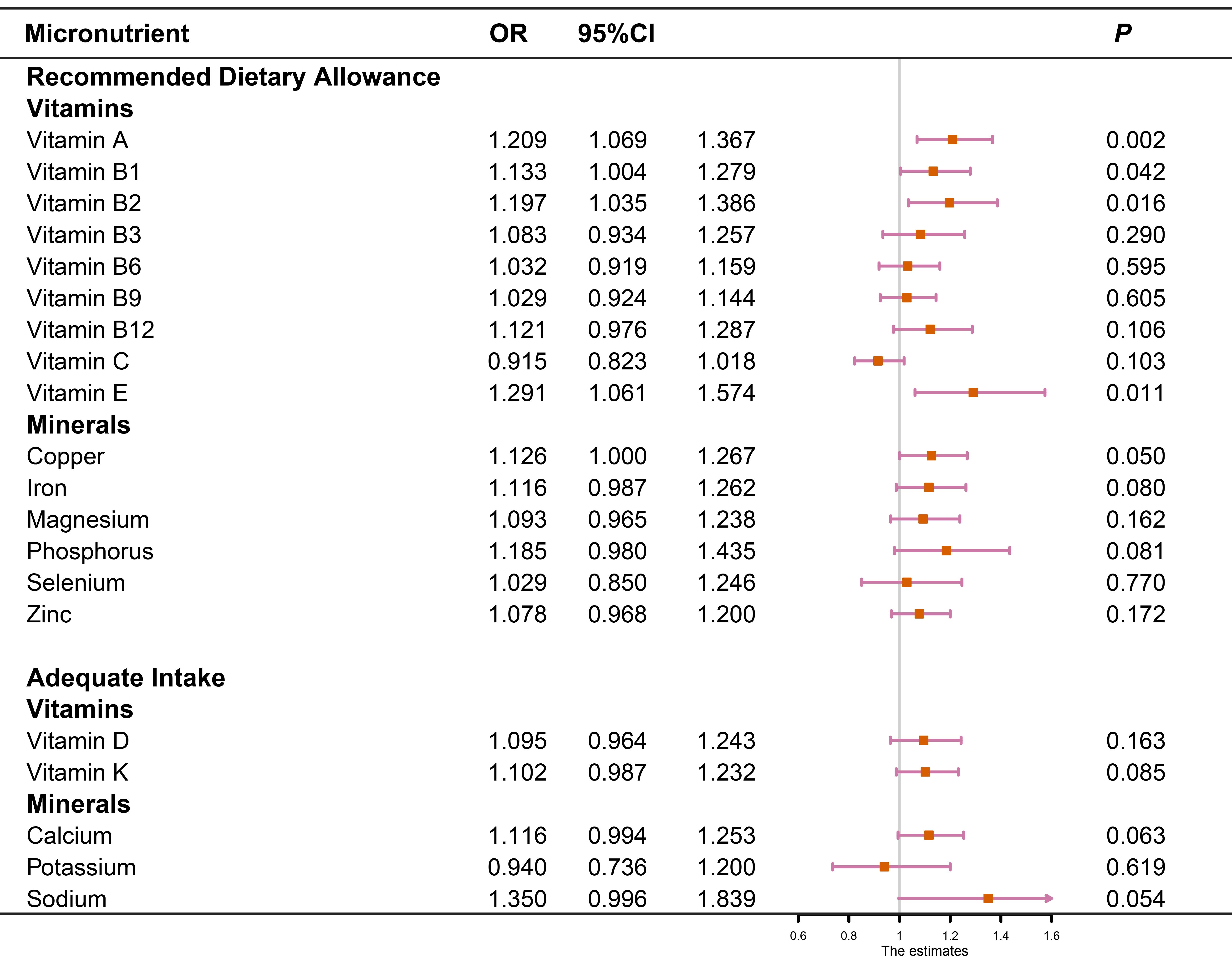

Supplement: Supplementary file 1 [file nutrients-14-02466-s001.zip › Figure S2.jpg]
